# Polydopamine-Pd nanozymes as potent ROS scavengers in combination with near-infrared irradiation for osteoarthritis treatment

**DOI:** 10.1016/j.isci.2023.106605

**Published:** 2023-04-18

**Authors:** Hao Hu, Junxu Yang, Yanping Zhong, Jiawei Wang, Jinhong Cai, Cuijuan Luo, Zhiqiang Jin, Ming Gao, Maolin He, Li Zheng

**Affiliations:** 1 Guangxi Engineering Center in Biomedical Materials for Tissue and Organ Regeneration, The First Affiliated Hospital of Guangxi Medical University, Nanning 530021, China; 2 Department of Spine Osteopathia, The First Affiliated Hospital of Guangxi Medical University, Nanning 530021, China; 3 Collaborative Innovation Center of Regenerative Medicine and Medical Bioresource Development and Application Co-constructed by the Province and Ministry, The First Affiliated Hospital of Guangxi Medical University, Nanning 530021, China; 4 Life Sciences Institute of Guangxi Medical University, Nanning 530021, China; 5 Department of Orthopaedics Trauma and Hand Surgery, The First Affiliated Hospital of Guangxi Medical University, Nanning 530021, China; 6 Guangxi Key Laboratory of Regenerative Medicine, The First Affiliated Hospital of Guangxi Medical University, Nanning 530021, China

**Keywords:** Biomedical materials, Health technology

## Abstract

Excessive reactive oxygen species (ROS) in joints could lead to gradual degeneration of the extracellular matrix (ECM) and apoptosis of chondrocytes, contributing to the occurrence and development of osteoarthritis (OA). Mimicking natural enzymes, polydopamine (PDA)-based nanozymes showed great potential in treating various inflammatory diseases. In this work, PDA loaded with ultra-small palladium (PDA-Pd) nanoparticles (NPs) was employed to scavenge ROS for OA therapy. As a result, PDA-Pd effectively declined the intracellular ROS levels and exhibited efficient antioxidative and anti-inflammatory capacity with good biocompatibility in IL-1β stimulated chondrocytes. Significantly, assisted with near-infrared (NIR) irradiation, its therapeutic effect was further enhanced. Further, NIR-stimulated PDA-Pd suppressed the progression of OA after intra-articular injection in the OA rat model. With favorable biocompatibility, PDA-Pd exhibits efficient antioxidative and anti-inflammatory capacity, leading to the alleviation of OA in rats. Our findings may provide new insights into the treatment of various ROS-induced inflammatory diseases.

## Highlights

• PDA-Pd NPs could scavenge various ROS efficiently
• NIR could significantly promote the catalase-like activity of PDA-Pd
• PDA-Pd exhibited efficient anti-inflammatory capacity *in vitro* and *in vivo*

## Graphical abstract

**Figure:**
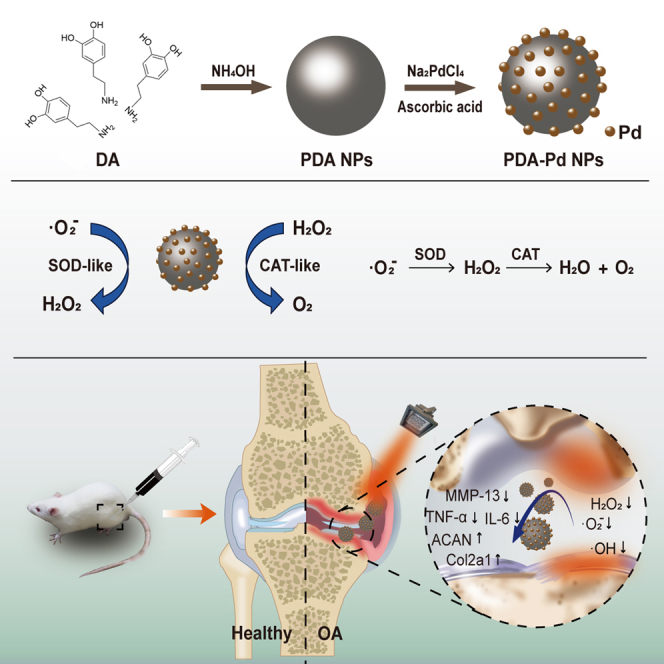

## Introduction

Osteoarthritis (OA), a low-grade inflammatory disease of joints, is prevalent worldwide with the rapid increase of the aging population.^1^ Nevertheless, effective treatments for OA are still scarce currently.^2^ Generally, OA is typically characterized by progressive damage to articular cartilage including the death of chondrocytes and the degradation of the extracellular matrix. This is mainly attributed to homeostasis dysregulation of the articular cartilage microenvironment, mainly caused by the accumulated excessive reactive oxygen species, such as hydroxyl radical (·OH), singlet oxygen (^1^O_2_), superoxide anion radical (·O_2_^−^), and hydrogen peroxide (H_2_O_2_) in the joint.^3^^,^^4^^,^^5^ Excessive ROS also can stimulate the production of matrix metalloproteinases (MMPs) in chondrocytes, resulting in progressive loss of articular cartilage.^3^ In view of the crucial role of ROS in the occurrence and progression of OA, ROS scavenging is possibly to be an effective strategy for OA therapy.

Increased evidence confirmed that polydopamine (PDA) could be applied to treat inflammatory diseases caused by excessive ROS via ROS scavenging.^6^ PDA, as one of the best antioxidants, is fabricated by the simple oxidation self-assembly of dopamine (DA) under alkaline conditions. It has been applied in the biomedical field widely because of its unique biocompatibility, biodegradability, high loading capacity of varied components, and outstanding NIR absorbance.^7^ Meanwhile, PDA contains a large number of reducing functional groups like catechol and imine that has been confirmed as excellent multifunctional ROS scavenger for alleviating ROS-induced diseases. Xingfu Bao et al. confirmed that PDA has a good effect of scavenging hydroxyl radicals and superoxide anions, and successfully used it to treat periodontitis in rats.^8^ Xiaotong Lou et al. confirmed that PDA can scavenge superoxide anions and hydroxyl radicals and can treat retinal ganglion cell degeneration after nerve injury by scavenging ROS.^9^ However, PDA has a limited scavenging effect. And it was easy to be cleared, which discounted its therapeutic effects. Combination with other materials may increase the bioavailability and the scavenging effects.^10^

In recent years, palladium-based nanozymes have attracted lots of attention in the biomedicine field attributed to their high-efficient multifunctional catalytic activities and photosensitive properties.^11^^,^^12^ Cuicui Ge et al. confirmed that palladium (Pd) nanoparticles have good catalase-like (CAT-like) enzyme and superoxide dismutase-like (SOD-like) enzyme activities.^13^ Zhi Jia et al. confirmed that Pd nanoparticles can scavenge hydroxyl radicals and superoxide anions.^14^ Yue Yang et al. also confirmed that Pd has a CAT-like enzyme effect. Significantly, Using NIR to irradiate Pd can trigger the localized surface plasmon resonance (LSPR) effect of Pd nanoparticles, which in turn generates hot electrons, resulting in enhanced CAT-like enzyme activity.^15^ Thus, the addition of Pd to PDA may enhance the ROS-scavenging effects of PDA.

Inspired by the above, we have developed a novel nanozyme (PDA-Pd) by loading ultra-small Pd NPs on the nanosized PDA for ROS scavenging in OA therapy. The novel PDA-Pd with NIR irradiation presented enhanced ROS-scavenging capacity *in vitro* and *in vivo*. Significantly, low cytotoxic PDA-Pd assisted by NIR effectively lowered the level of inflammatory factors and promoted the expression of chondrocyte-specific proteins, leading to the alleviation of OA in Sprague-Dawley (SD) rats. It offers a feasible strategy to develop novel nanozymes with a synergistic enhancement of ROS-scavenging effects for the prevention and therapy of ROS-related diseases.

## Results

### Synthesis and characterization of PDA and PDA-Pd NPs

**Figure 1:**
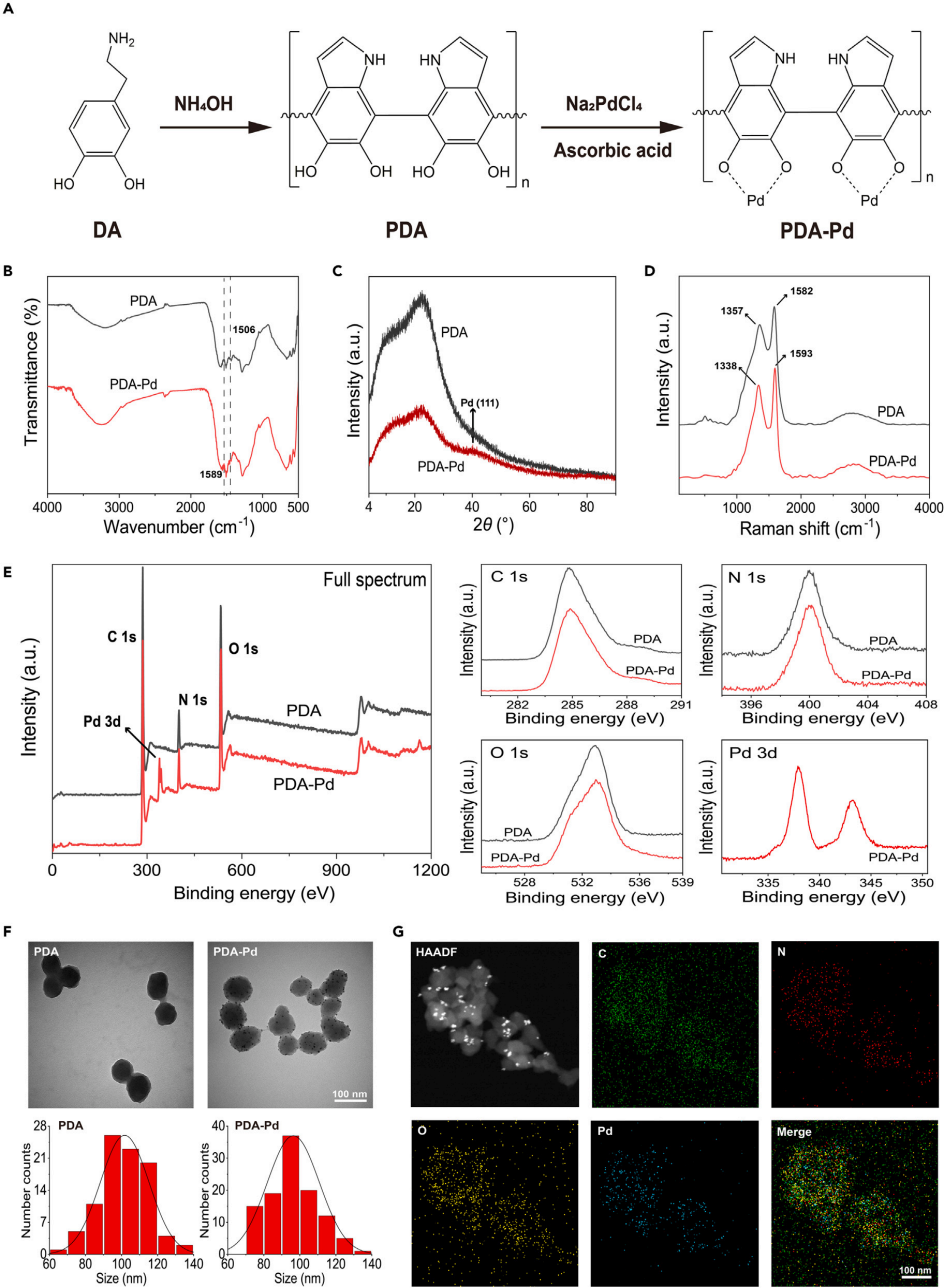
Preparation and characterizations of PDA and PDA-Pd (A) The chemical synthesis procedure of PDA-Pd. (B) FTIR results of PDA and PDA-Pd. (C) XRD results of PDA and PDA-Pd. (D) Raman spectra of NPs. (E) XPS results of NPs. (F) TEM images of PDA and PDA-Pd, and their corresponding statistical analysis of size distribution. (scale bar: 100 nm). (G) High angle annular dark field (HAADF) image and elemental mapping images of PDA-Pd. (scale bar: 100 nm). See also Figures S1–S5.

Due to their good biocompatibility and biodegradability, PDA and its derivatives had been widely applied in biomedical applications.^7^ First, PDA was prepared by a simple redox reaction of DA after adjusting to alkaline conditions with the addition of an ammonia solution. Subsequently, PDA was loaded with Pd NPs by the reduction of ascorbic acid to form PDA-Pd (Figure 1A). Next, the chemical structures of PDA and PDA-Pd were characterized via the Fourier transform infrared (FTIR) spectrum, ultraviolet-visible (UV- vis) spectra, and Raman spectra. As illustrated in Figure 1B, no apparent differences were observed between PDA and PDA-Pd by FTIR. And the UV-Vis spectra of PDA and PDA-Pd were also similar (Figure S1). The obvious Raman shift was found at 1357 and 1582 cm^−1^ for PDA. After Pd loading, the Raman shift changed to 1338 and 1593 cm^−1^ respectively (Figure 1D). And their crystallization and phase composition were also analyzed by X-ray diffraction (XRD). As shown in Figure 1C, a weak peak of 40.1° at 2θ values correspond to the diffraction from the (111) lattice plane of Pd was found in the XRD pattern of PDA-PD but not in the PDA pattern. This was explained by the loading of ultrafine Pd NPs on PDA NPs. Meanwhile, from the results of the X-Ray photoelectron spectrum (XPS), it was found that C, N, and O spectra happened to both PDA and PDA-Pd. However, the Pd spectrum was only observed for PDA-Pd (Figure 1E). Inductively coupled plasma-optical emission spectrometry (ICP-OES) illustrated the Pd content of 6.14% in the composite, which was quite different from the result of XPS. Besides, the surface morphology of PDA and PDA-Pd was characterized by scanning electron microscopy (SEM) and transmission electron microscopy (TEM). The obvious spherical structure was observed PDA and PDA-Pd in the SEM images of PDA and PDA-Pd (Figure S2). By TEM, it was also observed that the ultra-small Pd NPs (<10 nm) were homogeneously dispersed on the surface of PDA (Figure 1F). And PDA-Pd nanoparticle has rougher surface than PDA as shown in the three-dimensional images provided by atomic force microscopy (AFM) (Figure S3). After statistical analysis, the average diameter of PDA and PDA-Pd was 101.72 ± 5.09 nm and 96.41 ± 4.82 nm respectively. Furthermore, from the elemental mapping images results, the obvious C, N, O, and Pd elements existed for PDA-Pd (Figure 1G). Besides, as a result of the thermogravimetric analysis (TGA), the thermal stabilities of PDA and PDA-Pd were similar. (Figure S4). The zeta potential of PDA was −61.10 mV, which changed to −22.07 mV for PDA-Pd (Figure S5).

From the above, it gave the proof of successful preparation of PDA-Pd with the Pd loading ratio of 6.14%. And PDA loading with ultra-small Pd did not change the molecular structure, surface morphology, or average diameter of PDA significantly, only leading to the change of zeta potential and element composition observably.

### Stability and degradation property

**Figure 2:**
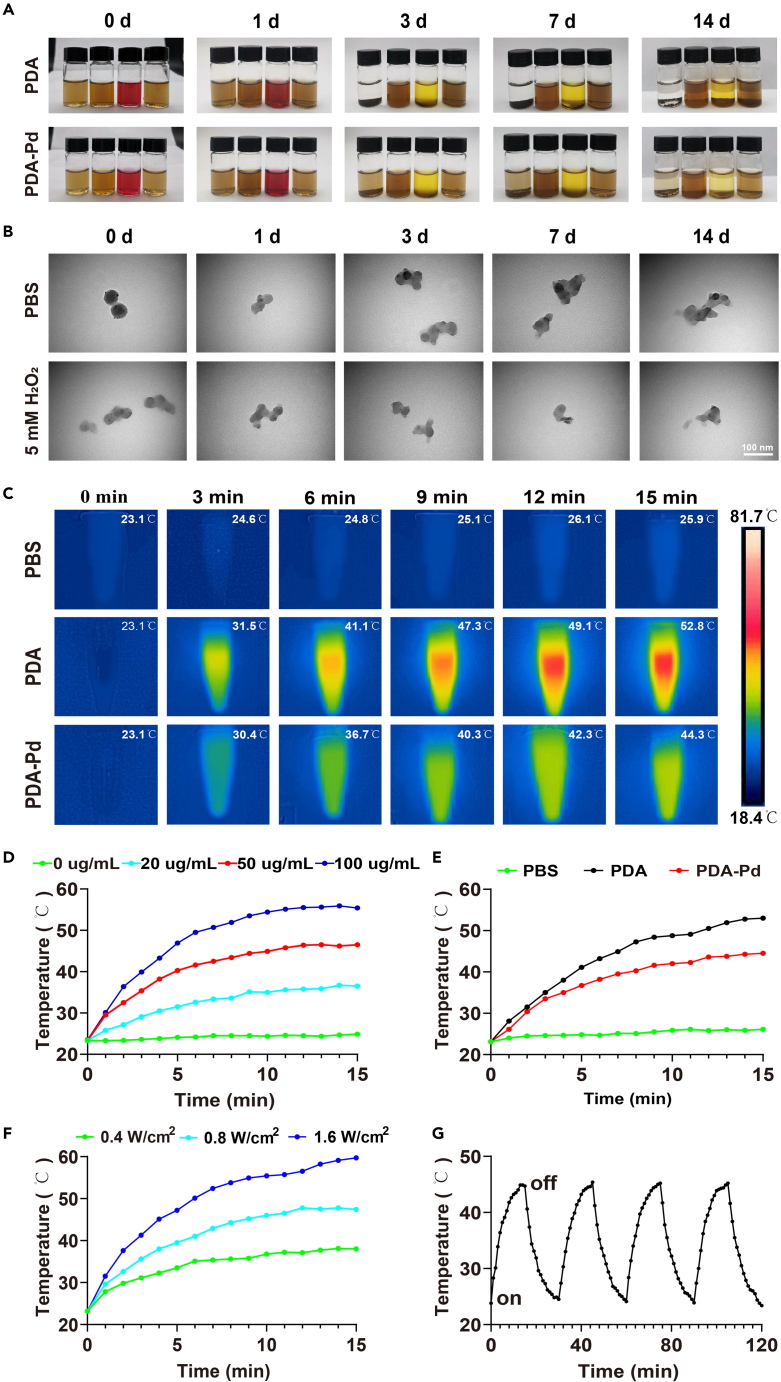
Physicochemical properties of PDA and PDA-Pd (A) Dispersibility and stability test of PDA and PDA-Pd in different solutions. (B) The degradability of PDA-Pd NPs. (C) *In vitro* photothermal images of PBS, PDA, and PDA-Pd. (D) Photothermal curves of PDA-Pd at different concentrations. (E) Photothermal curves of PBS, PDA, and PDA-Pd. (F) Photothermal curves of PDA-Pd with different power densities. (G) Photothermal stability test of PDA-Pd NPs during four on/off circles.

For practical application, it required that PDA-Pd NPs could retain a certain degree of stability and degradability in physiological conditions. Thus, PDA and PDA-Pd were respectively dispersed in PBS, fetal bovine serum (FBS), DMEM, and 5 mM H_2_O_2_ solutions for 14 days. As indicated in Figure 2A, PDA and PDA-Pd could maintain a certain degree of stability and were homogeneously distributed in the solutions for more than 1 day. On day 3, it was observed that PDA NPs had deposited at the bottom of PBS and DMEM solutions, whereas, quite a few of PDA-Pd NPs were still distributed in PBS on day 3. Furthermore, when it came to day 14, almost all PDA NPs had deposited on the bottom while only a few of PDA-Pd were observed on the bottom in the PBS buffer. Specifically, PDA and PDA-Pd were still well dispersed in the FBS buffer until the 14th day. In the meantime, PDA-Pd NPs at predetermined time points were also observed by TEM to evaluate their degradability. On day 7, it was observed that PDA-Pd NPs retained the spherical shape in PBS buffer while they started to degrade in 5 mM H_2_O_2_ solution with the damaged shapes (Figure 2B). The above results confirmed that PDA-Pd NPs could retain relative stability in various physiological conditions. Meanwhile, it also could degrade gradually as time goes by, especially in a 5 mM H_2_O_2_ solution.

### Photothermal behavior

Generally, NIR stimulation could be helpful to improve the unique function of nanocarriers, including accelerating the release of medicines and electron transfer reactions.^16^^,^^17^ PDA and PDA-Pd NPs were homogeneously dispersed in PBS buffer with different concentrations under NIR irradiation to test the photothermal behavior. Under 0.8 W/cm^2^ NIR irradiation, when increasing the concentration of PDA-Pd from 0 to 20, 50, and 100 μg/mL, the temperature jumped from 24.9°C to 36.5°C, 46.5°C, and 55.4°C within 15 min respectively (Figure 2D). As shown in Figure 2E, compared to the PBS buffer, the temperature of PDA and PDA-Pd increased with the extension of NIR irradiation time. After statistical analysis, for 50 μg/mL PDA alone, the temperature went up from room temperature to 53°C, where it only increased to 44.5°C for PDA-Pd after NIR irradiation. Meanwhile, the corresponding thermal images of PBS, PDA, and PDA-Pd were captured with the same concentration at different time points (Figure 2C). Compared to PDA, the slightly lower temperature existed for PDA-Pd, which was possibly attributed to the existence of ultra-small Pd NPs affected the photothermal effects. Similarly, increasing the power intensity from 0.4 to 1.6 W/cm^2^, the corresponding temperature increased from 38°C to 59.7°C (Figure 2F). Significantly, after 4 cycles, the photothermal properties of PDA-Pd still maintained relatively stable (Figure 2G). It confirmed that PDA-Pd NPs possessed good photothermal effects and photothermal stability. Increasing the concentration and power intensity contributed to a stronger photothermal effect.

### ROS-scavenging capacity

**Figure 3:**
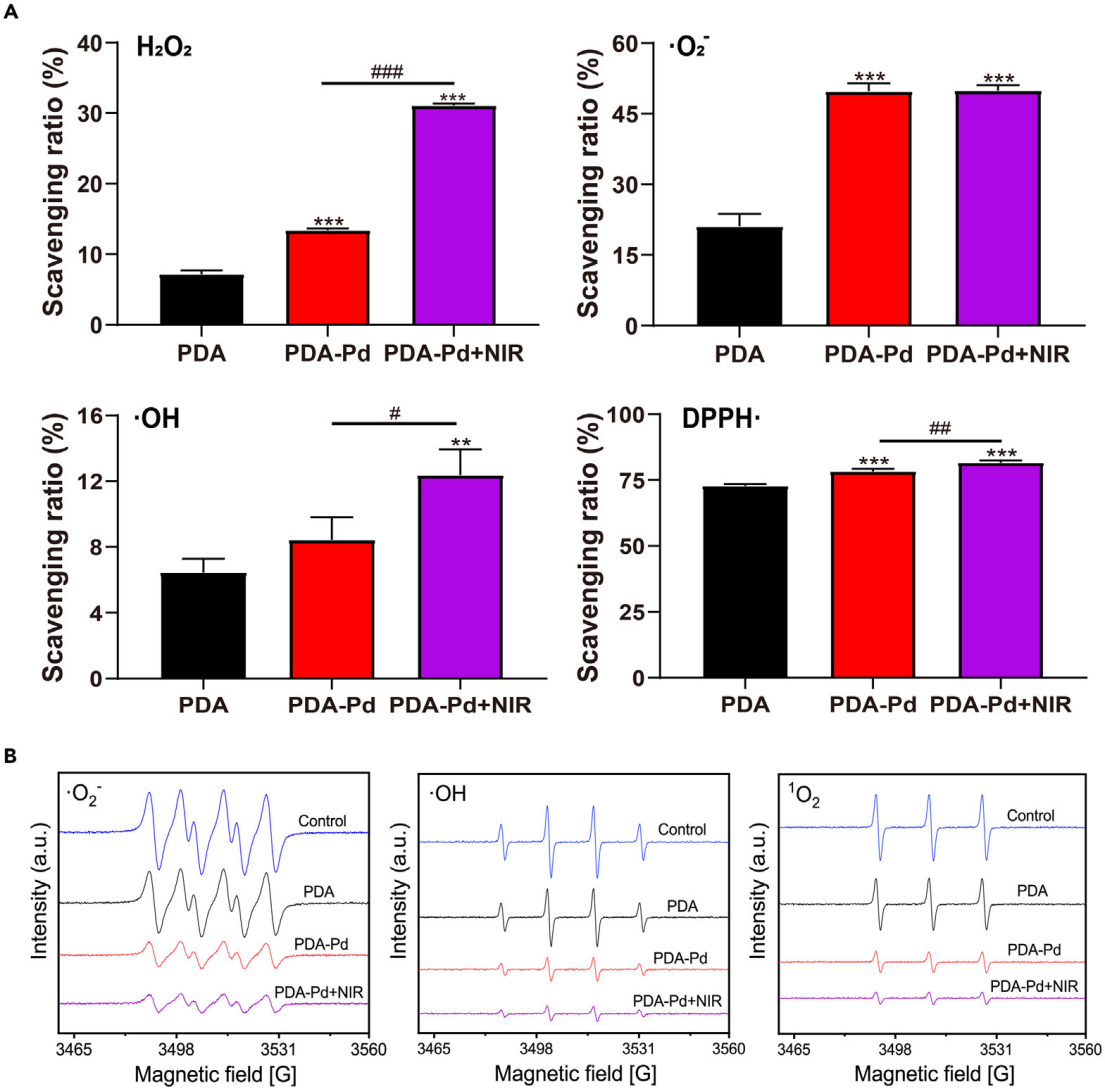
ROS-scavenging capacity of PDA, PDA-Pd, and PDA-Pd with NIR irradiation (A) H_2_O_2_, ·O_2_^−^, ·OH, and DPPH· scavenging ability measured by ROS detection kit. (B) O_2_^−^, OH, and ^1^O_2_ scavenging ability measured by ESR. See also Figures S6 and S7.

The ROS-scavenging capacity was initially evaluated using ROS detection kits. As shown in Figure 3A, PDA presented a certain degree of the scavenging capacity of ·O_2_^−^, H_2_O_2_, ·OH, and DPPH· with the scavenging ratio of 21.05%, 7.14%, 6.44%, and 72.78% detected by ROS detection kits respectively. After Pd NPs loading, the corresponding scavenging effects increased to 49.79%, 13.37%, 8.42%, and 78.25% for PDA-Pd. With NIR irradiation the H_2_O_2_ scavenging capacity of PDA-Pd significantly increased from 13.37% to 31.09% compared to without NIR. And NIR irradiation could promote the ·OH scavenging capacity from 8.42% to 12.36%, while the ability to scavenge DPPH · was only slightly increased, from 78.25% to 81.64%. Conversely, NIR irradiation did not improve the ·O_2_^−^ scavenging ability of PDA-Pd.

And the ROS-scavenging capacity was also concentration dependent. As indicated in Figure S6, it was observed that increasing the concentration of PDA-Pd could be equal to the improved scavenging capacity of ·O2-, ·OH, H_2_O_2_, and DPPH free radical.

Besides, we also determined the total ROS-scavenging ability by ROS detection kits. As indicated in Figure S7, the best scavenging ability of total ROS was found when PDA-Pd NPs were added with NIR irradiation (Figure S7A). And increasing the concentration of PDA-Pd could improve the scavenging capacity of total ROS (Figure S7B).

At last, the ROS-scavenging capacity was also assessed by electron spin resonance (ESR). As indicated in Figure 3B, compared to the control group without any treatments, the ESR signals of ·O_2_^−^, ·OH, and ^1^O_2_ were weakened by the treatment of PDA NPs and became weaker by the treatment of PDA-Pd. The weakest signals of ROS were found when PDA-Pd NPs were added with NIR irradiation.

From the above, PDA and PDA-Pd NPs possessed the obvious ROS-scavenging capacity. Specifically, the most effective ROS-scavenging capacity happened to PDA-Pd with NIR irradiation. With NIR irradiation, it could be helpful to accelerate the catalytic reaction of metals, leading to ROS-scavenging capacity with high efficacy.

### Cell viability

**Figure 4:**
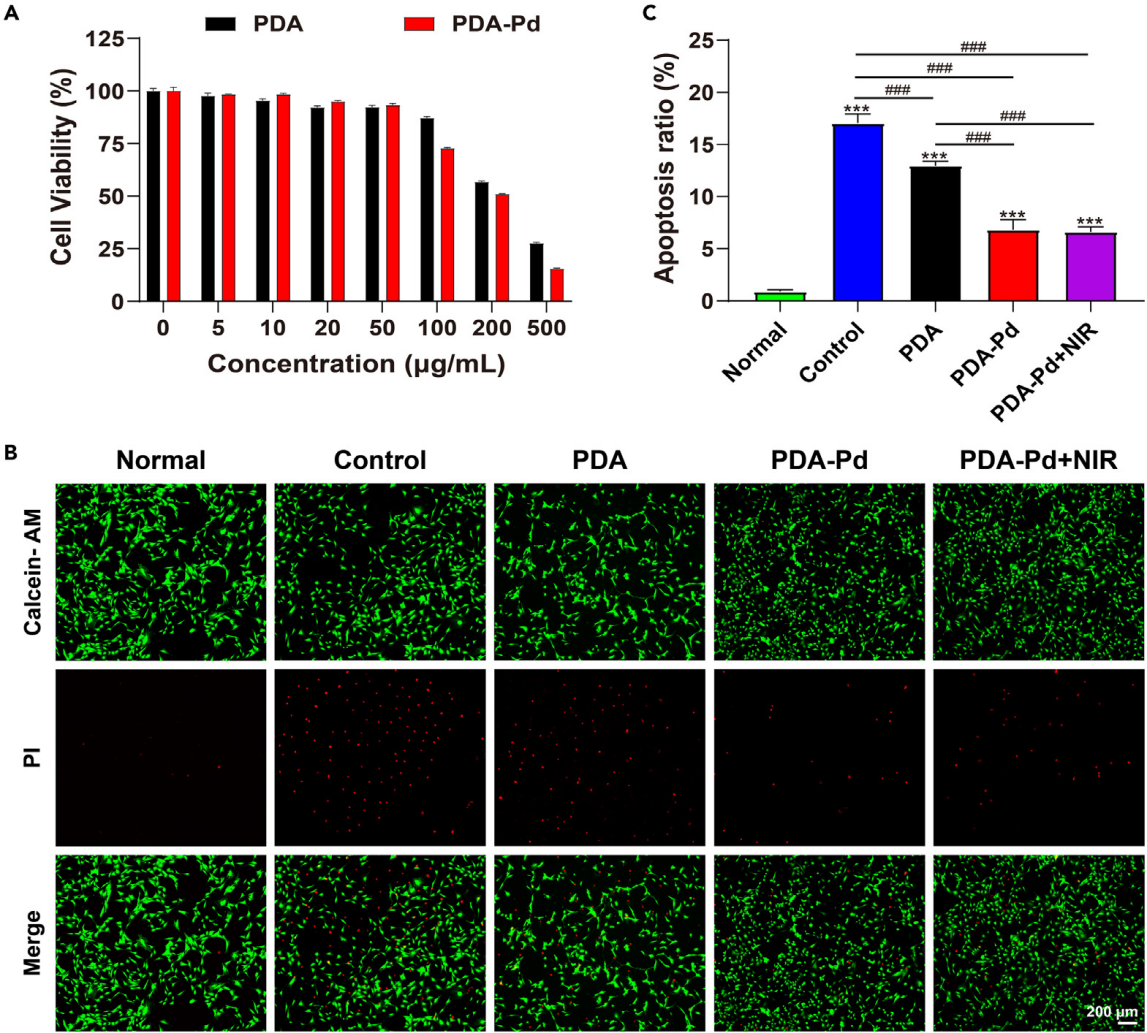
The cytotoxicity of NPs and the protection of NPs toward cells (A) Relative viability of chondrocytes after incubation with PDA and PDA-Pd at different concentrations. (B) Fluorescence images of chondrocytes under different treatment conditions, stained with calcein-AM and PI. Scale bar = 200 μm. (C) Quantification of relative apoptosis ratio of chondrocytes under different treatment conditions by ImageJ software, after staining with calcein AM/PI. (n = 3, values are the means ± SD. ∗ symbol is for the comparison between the normal group and every other group, ∗ and ^#^ symbol is for the pairwise comparison. ∗ and ^#^ indicates p < 0.05; ∗∗ and ^##^ indicates p < 0.01; ∗∗∗ and ^###^ indicates p < 0.001).

For biomedical applications, it was expected that PDA-Pd NPs possessed good biocompatibility. From the results of the cell counting kit-8 (CCK-8) kit, it displayed that PDA and PDA-Pd had favorable biocompatibility below 50 μg/mL with cell viability above 90% (Figure 4A). PDA has a similar structure and chemical properties to melanin, a natural biopolymer, with low cytotoxicity and good biocompatibility.^11^^,^^18^ And multiple previous experiments have confirmed the low cytotoxicity of Pd.^13^^,^^19^^,^^20^ Combined with previous studies as well as the results of this experiment, we confirmed that PDA-Pd has low cytotoxicity.

In the meantime, the protective effects of PDA and PDA-Pd NPs were explored by live/dead staining. As indicated in Figure 4B, for the normal chondrocytes, few dead cells (red) were observed and lots of live cells were observed. After IL-1β stimulation, a lot of dead cells existed. The incubation of PDA and PDA-Pd NPs could effectively protect the chondrocytes avoiding the toxicity of ROS with the increased number of live chondrocytes. After statistical analysis, the percentage of dead chondrocytes was 17.05% for IL-1β stimulated chondrocytes; it decreased to 12.96%, 6.82%, and 6.62% respectively for PDA, PDA-Pd, and PDA-Pd with NIR irradiation treatment (Figure 4C). It confirmed that PDA-Pd NPs could effectively improve the viability of IL-1β-induced chondrocytes. Specifically, PDA-Pd and PDA-Pd + NIR irradiation presented the best protection effects. The protective effect of PDA-Pd with or without NIR irradiation on chondrocytes was not statistically different.

### ROS-scavenging and anti-inflammatory ability

**Figure 5:**
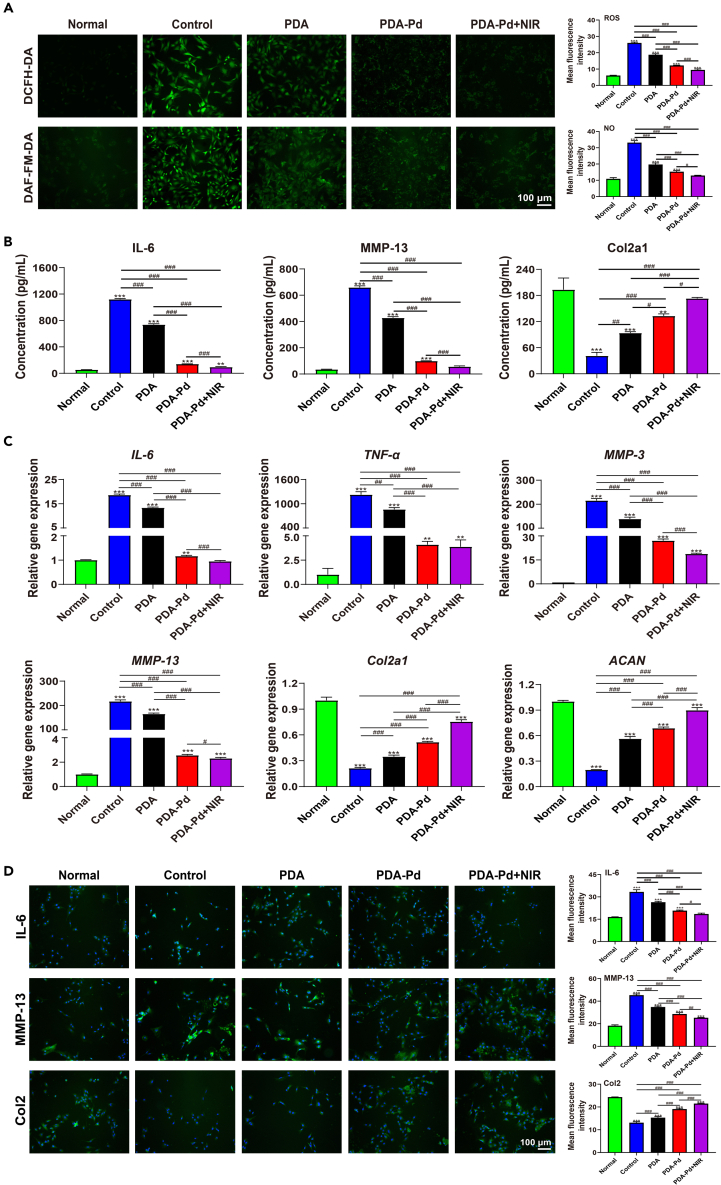
ROS-scavenging and anti-inflammatory capacity at the cellular levels (A) ROS staining (DCFH-DA) and RNS staining (DAF-FM-DA) of chondrocytes under different treatments. And quantification of mean fluorescence intensity in chondrocytes. (B) Detection of various proteins in the supernatant of chondrocytes under different treatments by ELISA. (C) Relative gene expressions in chondrocytes were determined by qRT-PCR. (D) Detection of various proteins in chondrocytes by immunofluorescence staining. And quantification of the mean fluorescence intensity in chondrocytes. Scale bar: 100 μm. (n = 3, values are the means ± SD. ∗ symbol is for the comparison between the normal group and every other group, and ^#^ symbol is for the pairwise comparison. ∗ and ^#^ indicates p < 0.05; ∗∗ and ^##^ indicates p < 0.01; ∗∗∗ and ^###^ indicates p < 0.001). See also Figure S9.

The ROS-scavenging capacities of PDA and PDA-Pd NPs at cellular levels were evaluated using the reactive nitrogen species (RNS) and ROS probes. Compared with normal chondrocytes, the total ROS levels significantly increased for IL-1β-induced chondrocytes (control group) with high-intensity green fluorescence over a large area observed. PDA and PDA-Pd NPs could decrease the total ROS levels significantly. Particularly, the fluorescence intensity decreased more obviously for PDA-Pd with NIR irradiation than that for PDA-Pd alone. It displayed the same tendency for RNS levels where the strongest fluorescence intensity was the control group and PDA-Pd+NIR possessed the lowest fluorescence intensity. After statistical calculation, the mean fluorescence intensity of total ROS levels was 6.01 for the normal chondrocytes, increased to 25.94 for the control group while it became 18.73 for PDA, 12.17 for PDA-Pd, and 9.46 for PDA-Pd+NIR respectively. Similarly, the mean fluorescence intensity for RNS was in the order of the control group> PDA> PDA-Pd> PDA-Pd+NIR (Figure 5A).

The scavenging capacity of hydroxy radicals of PDA and PDA-Pd NPs at cellular levels was evaluated using the hydroxyl radical detection kit (hydroxyphenyl fluorescein, Maokangbio, China). Compared with normal chondrocytes, the hydroxyl radical levels significantly increased for IL-1β-induced chondrocytes (control group) with high-intensity green fluorescence over a large area observed. PDA and PDA-Pd NPs could decrease the hydroxyl radical levels significantly (Figure S9).

The levels of inflammatory cytokines (MMP-13 and IL-6) and chondrocyte-specific protein (Col2a1) of cell supernatant were analyzed by ELISA. As indicated in Figure 5B, the protein level of IL-6 was super low (53.73 pg/mL) for the normal chondrocytes, and significantly jumped to 1120.10 pg/mL for the control group. After treatment, it was 736.32 pg/mL for PDA and 140.40 pg/mL for PDA-Pd respectively. Specifically, it was 94.11 pg/mL for PDA-Pd with NIR irradiation. Similar results were obtained for MMP-13, the highest expression levels happened to the control group while PDA-Pd+NIR possessed the optimum downregulation effects. Conversely, the expression of Col2a1 was low with a value of 41.33 pg/mL for the control group, which increased to 93.80, 132.91, and 173.08 for PDA, PDA-Pd, and PDA-Pd+NIR, respectively.

The gene expression situation was assessed by qRT-PCR (The genes and the corresponding primer sequences were shown in Table 1). From Figure 5C, compared to normal chondrocytes, the levels of *TNF-α*, *IL-6*, *MMP-13*, and *MMP-3* significantly ascended for chondrocytes after IL-1β induction. However, PDA and PDA-Pd NPs could descend the levels of inflammatory cytokines, especially for PDA-Pd NPs. Significantly, under NIR irradiation, it could more effectively decrease the gene expression of *IL-6*, *MMP-3*, and *MMP-13*. Besides, the chondrocyte-specific genes (*ACAN* and *Col2a1*) went up by PDA and PDA-Pd NPs treatment compared to the control group, especially for PDA-Pd NPs. And the growing trend became more obvious for PDA-Pd with NIR irradiation.

**Table 1:** Detailed primer sequences of qRT-PCR

Gene	Forward primer	Reverse primer
*GAPDH*	TCCAGTATGACTCTACCCACG	CACGACATACTCAGCACCAG
*TNF-α*	GATCGGTCCCAACAAGGAGG	GCTTGGTGGTTTGCTACGAC
*MMP-13*	ACCATCCTGTGACTCTTGCG	TTCACCCACATCAGGCACTC
*IL-6*	ACAAGTCCGGAGAGGAGACT	ACAGTGCATCATCGCTGTTC
*ACAN*	GAATGGGAGCCAGCCTACAC	GAGAGGCAGAGGGACTTTCG
*Col2a1*	GACTGTGCCTCGGAAGAACT	TCTGGACGTTAGCGGTGTTG
*MMP-3*	GGCTGTGTGCTCATCCTACC	TGGAAAGGTACTGAAGCCACC

Finally, the protein levels of inflammatory cytokines (IL-6 and MMP-13) and chondrocyte-specific protein (Col2) were also characterized by immunofluorescence staining. After IL-1β stimulation, the levels of IL-6 and MMP-13 were high while they declined after PDA and PDA-Pd NPs treatment. By statistical calculation, the mean fluorescence intensity of IL-6 was in the order of control group> PDA> PDA-Pd> PDA-Pd+NIR, similar to the tendency of mean fluorescence intensity of MMP-13. The expression levels of Col2 decreased significantly after IL-1β stimulation in chondrocytes, however, PDA treatment significantly increased the expression of Col2, and PDA-Pd could further improve the therapeutic effect, especially after NIR irradiation (Figure 5D).

From the above, it gave proof that PDA-Pd NPs could effectively downregulate the expression of inflammatory cytokines and upregulate the level of chondrocyte-specific proteins due to their excellent anti-inflammation and ROS-scavenging capacity. Among them, the optimum therapeutic effects happened to PDA-Pd with NIR irradiation.

### *In vivo* therapy effect

**Figure 6:**
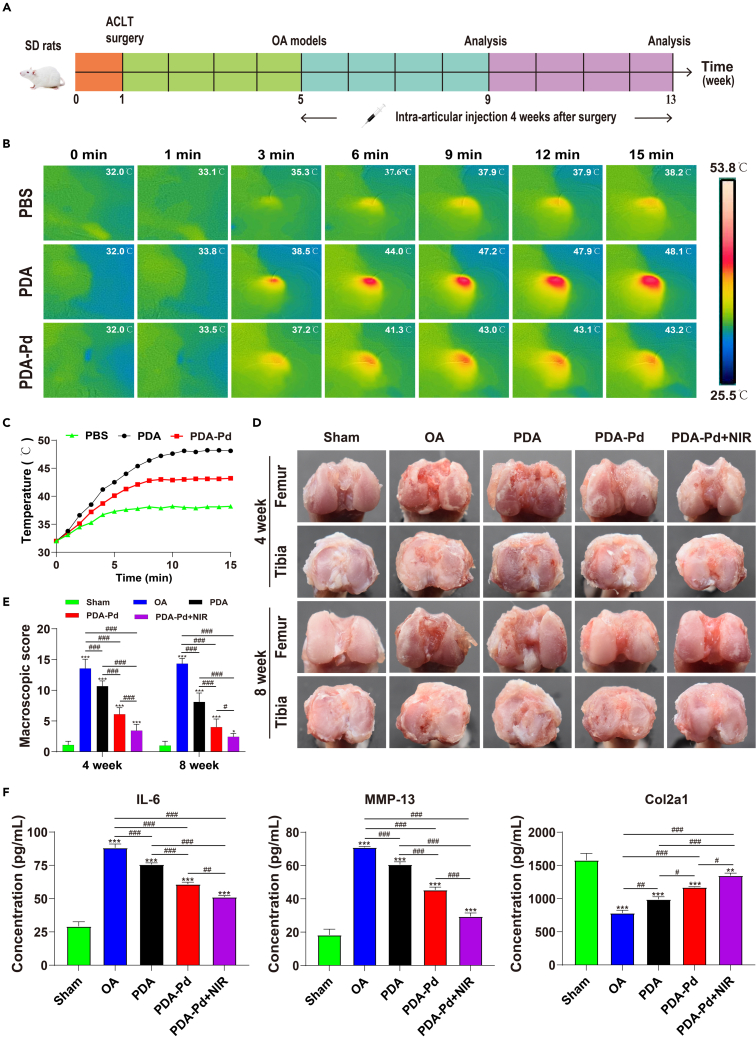
*In vivo* treatment effects of NPs (A) The schedule of the animal experiments. (B) *In vivo* photothermal images of PBS, PDA, and PDA-Pd. (C) *In vivo* photothermal curves of PBS, PDA, and PDA-Pd. (D) Macroscopic appearance of the articular surface. (E) The severity of OA was scored by a macroscopic scoring system. (F) Detection of various proteins in synovial fluid by ELISA. (Values are the means ± SD. ∗ symbol is for the comparison between the normal group and every other group, and ^#^ symbol is for the pairwise comparison. ∗ and ^#^ indicates p < 0.05; ∗∗ and ^##^ indicates p < 0.01; ∗∗∗ and ^###^ indicates p < 0.001).

The *in vivo* OA therapy was implemented by PDA and PDA-Pd NPs intra-articular injection with or without NIR irradiation. First, we explored the photothermal effects of NPs *in vivo*. As shown in Figures 6B and 6C, it was found that the temperature increased versus NIR irradiation time for SD rats with PDA or PDA-Pd injection. Compared to PBS injection, the equilibrium temperature jumped from 38.2°C to 48.1°C for PDA injection while it changed to 43.2°C for PDA-Pd injection during NIR irradiation. Therefore, it offered the feasibility of *in vivo* photothermal OA therapy.

Meanwhile, the macroscopic observation of joints was shown in Figure 6D. For the OA group, the degree of inflammation gradually became intensified with a rough and erosive surface of joints as time went on. Nevertheless, it had an obvious therapeutic effect after PDA and PDA-Pd NPs intra-articular injection, with a significant reduction of deterioration. Specifically, PDA-Pd with NIR irradiation significantly reduced the degree of inflammation with almost no defect and erosion existing, close to the normal joint at week 8. After macroscopic scoring based on Pelletier’s methods, it was 1.1 and 1.0 for the sham group while it increased to 13.6 and 14.3 for the OA group at week 4 and week 8 respectively. For PDA-Pd+NIR, the scores significantly decreased to 3.4 at week 4 and 2.4 at week 8 (Figure 6E).

To evaluate the OA therapy effect, the proteins in synovial fluid after treatment were analyzed by ELISA. As illustrated in Figure 6F, the level of IL-6 and MMP-13 in synovial fluid was relatively low for the sham group while it was at a high level for the OA group. After PDA and PDA-Pd NPs treatment, the expression of inflammatory factors declined. Specifically, for PDA-Pd with NIR irradiation, the downtrend became more significant. Conversely, the expression of Col2a1 had significantly improved after PDA and PDA-Pd NPs treatment. Among them, PDA-Pd with NIR irradiation possessed the best improvement of the expression of Col2a1.

**Figure 7:**
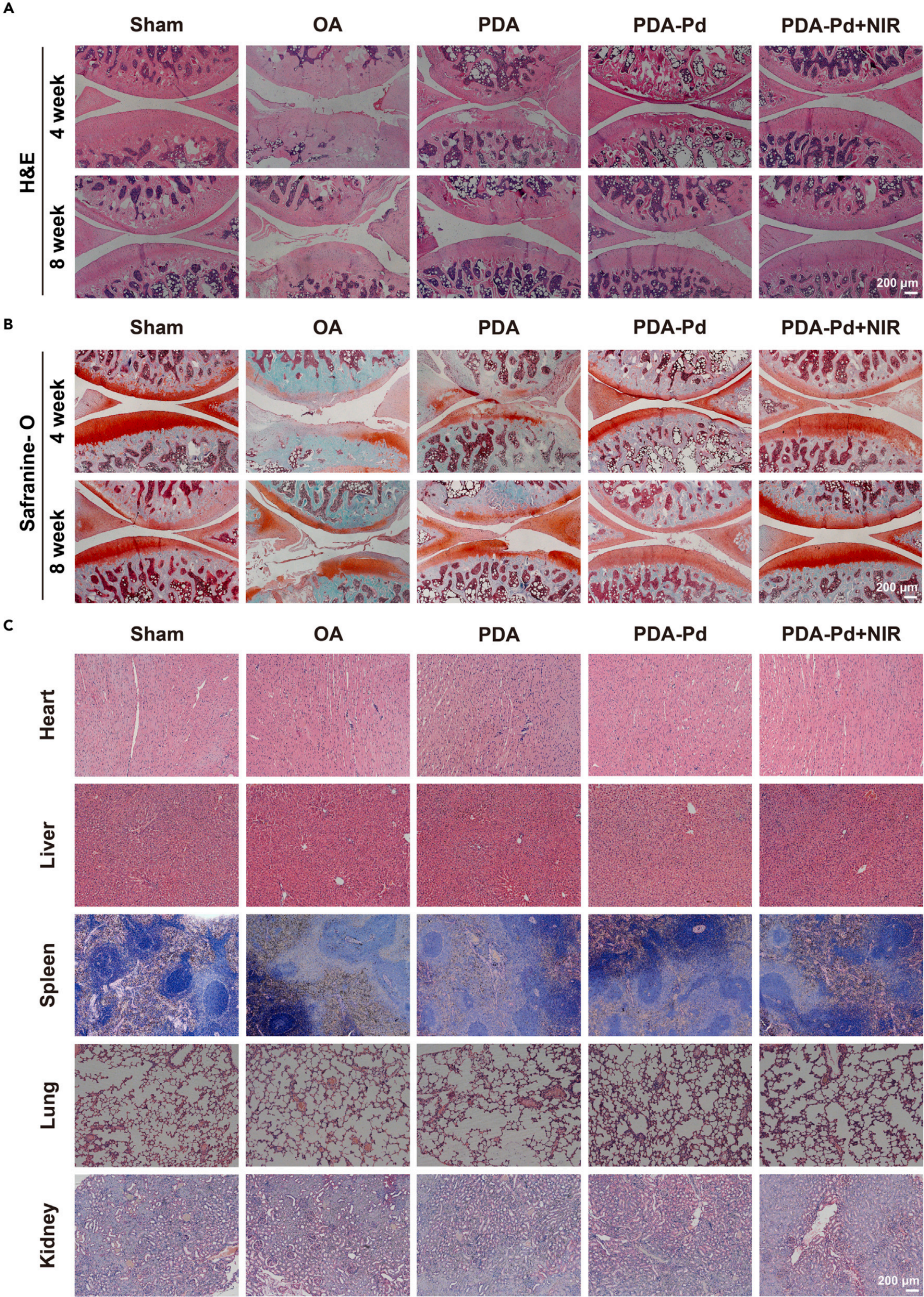
Histological evaluation of OA therapy (A) H&E staining images of joints. (B) Safranin O staining images of joints. (C) H&E staining histological images of the major organs. See also Figures S8, S10, and S11.

Besides, the results of histological staining were indicated in Figures 7A and 7B. For H&E staining compared to the sham group, obvious OA features like matrix loss, fissures, and fibrosis existed for the OA group at week 4 and week 8. For PDA, it was observed that the matrix loss was slightly restored. Significantly, the obvious restoration of matrix happened to PDA-Pd, especially for PDA-Pd+NIR at week 4 and week 8 (Figure 7A). Similarly, for Safranin O staining, the destroyed cartilage layers happened to the OA group while it was partially restored after PDA and PDA-Pd NPs treatment. Specifically for PDA-Pd+NIR, the repaired thick cartilage layer was observed infinitely close to the sham group (Figure 7B). In order to quantitatively assess the degree of cartilage inflammation, we also did the cartilage pathological scoring based on the Osteoarthritis Research Society International (OARSI) scoring system.^21^ It was 1.00 and 1.33 for the sham group while it increased to 5.22 and 5.67 for the OA group at week 4 and week 8 respectively. For PDA-Pd+NIR, the scores significantly decreased to 2.67 at week 4 and 2.22 at week 8 (Figure S8).

Finally, to evaluate the toxicity of PDA and PDA-Pd *in vivo*, the major organs were collected after intra-articular injection for 8 weeks. As indicated in Figure 7C, the H&E images of all organs of different groups presented almost the same conditions. It confirmed that it had no significant cytotoxicity for PDA and PDA-Pd NPs intra-articular injection with or without NIR irradiation. And we also completed the blood routine test and blood biochemistry test to study the toxicity of NPs. As shown in Figure S10, there was no significant difference in red blood cell count (RBC), hemoglobin (HGB), hematocrit (HCT), platelet count (PLT), and white blood cell count (WBC) among the four groups in the blood routine test. As indicated in Figure S11, there was no significant difference in the results of aspartate aminotransferase (AST), alanine aminotransferase (ALT), creatinine (CREA), and urea among the four groups. In our study, PDA and PDA-Pd NPs were administered locally in the joint at a quite low dose of 10 μg/rat each time, and no significant toxicity was observed.

## Discussion

ROS plays an important role in the occurrence and progression of OA.^22^ ROS-scavenging has emerged as an important strategy for treating OA.^23^ Among the numerous ROS-scavenging materials, PDA has attracted lots of attention due to its excellent ROS-scavenging ability and biocompatibility. However, PDA, as an antioxidant, also shares some common drawbacks of antioxidants. Antioxidants scavenge ROS by direct redox reactions with ROS, and the content of their reducing groups will be gradually consumed with the clearance of ROS, so their effective therapeutic concentrations are difficult to maintain for a long time, and the frequently repeated administration is required, which brings great difficulties to its practical application. Contrary to antioxidants, nanozymes, a new type of catalytic nanomaterials developed in recent years, such as Fe_3_O_4_, Mn_3_O_4_, and CeO_2_, have quite long-lasting and efficient catalytic activity.^24^^,^^25^^,^^26^ But, these nanozymes are also difficult to be widely applied in biomedicine because of their inherent disadvantages of easy agglomeration and difficulty in entering cells.^27^

In view of the advantages and disadvantages of PDA and nanozymes, some scholars tried to combine PDA and nanozymes in order to obtain more perfect ROS-scavenging materials. Xinyue Guo et al. developed a complex nanozyme PDA@CeO_2_ to maintain normal cell morphology by scavenging ROS.^28^ Xiaochun Hu et al. established a nanozyme by combining Pt nanozymes and PDA that can overcome tumor hypoxia by decomposing H_2_O_2_.^29^ Recently, palladium nanomaterials have received much attention due to their excellent physicochemical stability and efficient multifunctional enzyme activity. In this study, to overcome the inherent shortcomings of PDA, we have developed a novel nanozyme by loading Pd onto PDA via a simple reduction reaction. The results of TEM-Mapping and XPS demonstrated that we successfully synthesized PDA-Pd NPs.

In the joint, there are various species of ROS, such as ·O_2_^−^, ^1^O_2_, ·OH, and H_2_O_2_. In order to treat OA, drugs should be able to scavenge different kinds of ROS. In this study, the ROS detection kits demonstrated that PDA-Pd had an excellent scavenging ability for H_2_O_2_, ·O_2_^−^, ·OH, and DPPH· and the results of ESR showed that PDA-Pd had excellent scavenging activity for ·O_2_^−^, ·OH, and ^1^O_2_.

NIR has gradually attracted much attention due to its excellent tissue-penetrating ability, excellent photothermal therapy, and photodynamic therapy effects. PDA, as an analog of melanin, has high light absorption in the NIR region.^7^ Palladium nanoparticles also have strong light absorption in the NIR region.^30^^,^^31^ As a conjugate of PDA and palladium, PDA-Pd theoretically should have good light absorption as well as the excellent photothermal effect in the NIR region. In this study, we found that PDA-Pd has a good photothermal effect both *in vitro* and *in vivo*.

Recently, some studies also found that NIR has good photocatalytic activity.^32^^,^^33^ In this study, both the ROS detection kit and ESR results confirmed that NIR could significantly promote the catalase-like activity of PDA-Pd. The anisotropy of the shape of the Pd nanoparticle shows strong localized surface plasmon resonance (LSPR) in the NIR region of the electromagnetic spectrum.^34^ Hot electrons generated by LSPR greatly promote the enzymatic activity of metal nanomaterials. Yue Yang et al. confirmed that Pd has a catalase enzyme-like effect. Using NIR to irradiate Pd can trigger the LSPR effect of Pd nanoparticles, which in turn generates hot electrons, resulting in enhanced CAT-like enzyme activity.^15^ Shanshan Li and coworkers also found that strained palladium nanosheets had CAT-like activity. Meanwhile, the CAT-like activity of strained palladium nanosheets can be further enhanced by NIR.^35^ And PDA can produce a synergistic effect with photocatalysts through π-π∗ electron transitions, thereby greatly enhancing the photocatalytic activity of the catalysts.^7^

From the above, it indicated that PDA-Pd+NIR irradiation possessed the optimum OA therapy effects. Previous research found that PDA had a certain degree of antioxidative function *in vitro* but was limited by its short effective treatment time *in vivo*. Therefore, PDA loading with Pd could be helpful to retain long-term therapy *in vivo*. NIR irradiation also accelerated the catalytic effect of PDA-Pd, further leading to OA therapy with high efficacy.

In summary, giving full-play efficient ROS-scavenging activity of nanozyems is of significance for suppressing OA progression. The novel PDA-Pd NPs were explored to act as nanozymes to alleviate OA. The results demonstrated that PDA-Pd could effectively scavenge ROS, downregulate the expression of inflammatory cytokines, and upregulate the expression of the chondrocyte-specific protein. With the addition of NIR irradiation, better therapeutic results were achieved. The excellent antioxidative ability and cartilage protection ability of PDA-Pd indicated a very effective method for suppressing OA progression, finally resulting in excellent OA therapeutic effects.

### Limitations of the study

There are many kinds of ROS in the inflammatory chondrocyte, such as hydroxyl radicals and superoxide anions. To explore the ability of PDA-Pd to scavenge ROS in the biological environment, we have done the ROS detection kit (DCFH-DA) and the hydroxyl radical detection kit on chondrocytes. Because of the limited funds and time, we have not done the experiment of superoxide anion detection kit at cellular levels. So the scavenging ability of the intracellular superoxide anion of PDA-Pd is not yet clear.

## STAR★Methods

### Key resources table

**Table:** 

REAGENT or RESOURCE	SOURCE	IDENTIFIER
**Antibodies**		

IL-6 polyclonal antibody	Proteintech, Wuhan, China	AB_11142677
MMP-13 polyclonal antibody	Proteintech, Wuhan, China	AB_2144858
Collagen Type Ⅱ polyclonal antibody	Proteintech, Wuhan, China	AB_2881147

**Chemicals and recombinant proteins**		

Dopamine hydrochloride	Aladdin (China)	
Ascorbic acid	Aladdin (China)	
Anhydrous ethanol	Sinopharm Chemical Reagent Co., Ltd. (China)	
Sodium tetrachloropalladate (Na_2_PdC_l4_)	Macklin (China)	
Hydrogen peroxide (H_2_O_2_, 30%)	Chongqing Chuandong Chemical Co., Ltd. (China)	
Recombinant Human IL-1b	Solarbio (China)	

### Resource availability

#### Lead contact

Requests and information for recourses should be directed to the lead contact, Maolin He (hemaolin@stu.gxmu.edu.cn).

#### Materials availability

This study did not generate new unique reagents.

### Method details

#### Materials

Dopamine hydrochloride (DA, > 98%) and ascorbic acid were provided by Aladdin (China). Anhydrous ethanol was purchased from Sinopharm Chemical Reagent Co., Ltd. (China). Sodium tetrachloropalladate (Na_2_PdCl_4_) was purchased from Macklin (China). Hydrogen peroxide (H_2_O_2_, 30%) and ammonium hydroxide (NH_4_OH, 25.0∼28.0%) were provided by Chongqing Chuandong Chemical Co., Ltd. (China). All chemicals were utilized directly without further purification.

#### Synthesis of PDA and PDA-Pd

PDA and PDA-Pd NPs were synthesized based on previously described work with slight modification.^36^^,^^37^ In brief, 160 mL anhydrous ethanol, 360 mL deionized (DI) water, and 12 mL NH_4_OH were added into a flask and stirred magnetically at room temperature. Then, 2g DA dissolved in DI water (40 mL) was added into the above mixture dropwise. Subsequently, the reaction lasted for 24 h before the black product was collected by centrifugation for 8 min at 11000 rpm. PDA NPs were finally obtained after vacuum drying. Subsequently, 100 mg PDA NPs were dispersed in 80 mL anhydrous ethanol and 6 mL DI water, following by the dropwise addition of 2 mL Na_2_PdCl_4_ aqueous solution (10 mg/mL). The mixture was magnetically stirred and reacted for 3 h before adding ascorbic acid solution (10 mg/mL, 6 mL) for another 2 h. The final product PDA-Pd was obtained after centrifugation and vacuum drying.

#### Basic characterization of PDA and PDA-Pd

To determine the chemical and molecular structures of PDA and PDA-Pd, the FTIR spectrum (IRAffinity-1S, Shimadzu, Japan), UV- vis spectra (UV-2700, Shimadzu, Japan), and Raman spectrum (Horiba, France) were applied respectively. The valence state, element composition, and content of nanoparticles were studied by XPS (Thermo ESCALAB 250Xi, Thermo Scientific, USA). The zeta potential of PDA and PDA-Pd was detected with a zeta sizer (Nano-ZS, Malvern, UK) in DI water. Additionally, the size, morphology, structure, and elementary composition of NPs were observed by TEM coupled with energy-dispersive X-ray spectroscopy (EDS) (FEI talos f200s, FEI, USA). Furthermore, the three-dimensional structure of PDA and PDA-Pd was evaluated by atomic force microscopy (AFM) on a multimode microscope (Bruker, Karlsruhe, Germany). In addition, to analyze the thermal properties of the nanoparticles, thermogravimetric analysis (TGA) was detected on a TGA instrument (TGA Q500) under N_2_ atmosphere using a TGA instrument (STD650, TA, USA). Finally, the crystallization and molecular structure of PDA and PDA-Pd NPs were measured by XRD on an X-ray diffractometer (Miniflex 600**,** Rigaku, Japan), and the detailed Pd content of PDA-Pd was analyzed by ICP-OES (Thermo, USA).

#### Photothermal effect investigation

To systematically assess the photothermal effects, PDA and PDA-Pd were placed under NIR irradiation. In brief, PDA-Pd NPs dispersed in PBS buffer (50 ug/mL) were irradiated by a NIR laser with varied power densities of 0.4, 0.8, and 1.6 W/cm^2^. Then PDA-Pd NPs were dispersed in PBS buffer with different concentrations (20, 50, 100 μg/mL), and then irradiated at 0.8 W/cm^2^ respectively. Subsequently, PBS buffer, PDA, and PDA-Pd NPs dispersed in PBS buffer (50 ug/mL) were respectively irradiated with the NIR laser (0.8 W/cm^2^). A FLIR NIR camera was applied to collect the corresponding temperatures. Specifically, for the“on and off” experiment, the PDA-Pd solution was irradiated for 15 min and cooled for 15 min. The above steps were repeated 4 times, and the corresponding temperatures were collected.

#### *In vitro* degradability

To investigate the stability and degradability, PDA and PDA-Pd were dispersed in PBS, FBS, DMEM (Thermofisher, China), and 5 mM H_2_O_2_ solution with the same concentration of 50 μg/mL respectively. At predefined time points, their dispersion conditions were imaged and their morphology was observed by TEM.

#### ROS scavenging capability

The ROS scavenging abilities of the nanoparticles were examined by the detection kit. Briefly, to detect the CAT-like activity, we first detected the H_2_O_2_ scavenging capability of PDA-Pd at different concentrations (20, 50, 100μg/mL) by a catalase assay kit (Beyotime, China), the absorbance at 520 nm was detected by a microplate reader (Thermo Scientific, USA). Following the same method, we detected the CAT-like activity of PDA, PDA-Pd, and PDA-Pd with NIR irradiation at a concentration of 50 μg/mL respectively, NIR irradiation was implemented at 0.8 W/cm^2^ for 5 min during incubation.

To detect the SOD-like activity, we detected the ·O_2_^-^ scavenging activity of PDA-Pd at different concentrations (20, 50, 100 μg/mL) by a total superoxide dismutase assay kit (Beyotime, China). Different concentrations of PDA-Pd NPs were added into the working solution. Then the absorbance at 450 nm was detected after standing for 30 min and calculated the ·O_2_^-^ scavenging ratio by comparing it with the blank. Following the same method, we detected the SOD-like activity of PDA, PDA-Pd, and PDA-Pd with NIR irradiation at a concentration of 50 μg/mL.

Besides, the absorbance at 515 nm, 536 nm, and 734 nm was respectively measured to investigate the DPPH free radical, ·OH, and total ROS scavenging capacity respectively following the similar above steps by a DPPH free radical scavenging ability detection kit (Solarbio, China), a micro hydroxyl free radical scavenging capacity assay kit (Solarbio, China) and total antioxidant capacity assay kit with ABTS method (Beyotime, China).

At last, the ROS scavenging ability was also evaluated by electron spin resonance (ESR, Bruker A300, Germany). In brief, after the working solution was treated with PDA, PDA-Pd, and PDA-Pd + NIR irradiation (0.8 W/cm^2^, 5 min) at the same concentration of 50 μg/mL respectively, the ESR signals of the residual ·OH, ·O_2_^-^, and ^1^O_2_ were measured.

#### Isolation and culture of chondrocytes

Chondrocytes were obtained from the femur and tibia of SD rats (3∼5 days old) as previously described.^38^ After extraction, the chondrocytes were cultured with DMEM containing 1% penicillin-streptomycin solution (Solarbio, China) and 10% FBS (Sijiqing, China). The chondrocytes were only passaged for two generations for further experimental procedures.

#### Cell viability

The cell viability was evaluated by CCK-8 (Biosharp, China). Briefly, chondrocytes were cultured in the 96-well plates with the density of 8000 chondrocytes per well. After 24 h of culture, the medium was replaced with fresh culture medium containing PDA or PDA-Pd with concentrations ranging from 0 to 500 μg/mL for another 24 h, each concentration was repeated 3 times. Subsequently, the chondrocytes were cultured with 100 μL medium and 10μL CCK-8 for 2 h after PBS buffer washing. Finally, the chondrocyte viability was quantified by detecting the absorbance at 450 nm.

In addition, the protective effect of PDA and PDA-Pd NPs on chondrocytes against apoptosis induced by IL-1β was also evaluated by live/dead staining. In detail, chondrocytes were seeded into 6-well plates with a density of 1× 10^5^ cells per well. After 24 h of culture, the chondrocytes were attached and the medium was removed. Subsequently, after IL-1β stimulation (10 ng/mL, 24 h), chondrocytes were incubated with PDA, PDA-Pd, or PDA-Pd plus NIR irradiation (0.8 W/cm^2^, 5 min) respectively at the same concentration of 50 μg/mL for 24 h. Then the wells were washed with PBS buffer twice. Then the chondrocytes were stained with Calcein AM/PI (Beyotime, China) for 5 min. Finally, the chondrocytes were imaged by a fluorescent microscope (Echo Revolve, China), and quantified by Image J software.

#### Intracellular ROS levels

The change of intracellular ROS and RNS levels of cells were examined by ROS detection kit (DCFH-DA, Beyotime, China), hydroxyl radical detection kit (hydroxyphenyl fluorescein, Maokangbio, China), and RNS detection kit (DAF-FM DA). Details were: chondrocytes were seeded into 6-well plates (2× 10^5^ cells per well), After 24 h of culture the chondrocytes were attached and the medium was removed. Next, after IL-1β stimulation (10 ng/mL, 24 h), chondrocytes were incubated with PDA, PDA-Pd, and PDA-Pd plus NIR irradiation (0.8 W/cm^2^, 5 min) respectively at the same concentration of 50 μg/mL for 24 h. Subsequently, the chondrocytes were respectively added with 10 μM 2',7'-dichlorodihydrofluorescein diacetate (DCFH-DA, Beyotime, China) for total ROS testing, 10 μM hydroxyphenyl fluorescein for hydroxy radical testing, and 5 μM 4-amino-5-methylamino-2',7'-difluoro-fluorescein (DAF-FM DA, Beyotime, China) for RNS testing for 20 min. At last, the chondrocytes were observed using a fluorescence microscope after washing with PBS buffer and the fluorescence intensity was analyzed using Image J software.

#### Quantitative real-time PCR

Chondrocytes were pretreated with 10 ng/mL IL-1β for 24 h and then exposed to various nanoparticles (50 μg/mL) for 24 h with or without NIR irradiation. Then the total RNA of chondrocytes was isolated by a HiPure total RNA mini kit (Magen, China). Then total RNA was reverse-transcribed into cDNA by a PrimeScript™ RT reagent Kit (Takara, Japan). Subsequently, qRT-PCR was completed with LightCycler® 96 System (Roche, Switzerland). The relative levels of gene expressions were analyzed via the 2−ΔΔCT method and normalized to *GAPDH*. The detailed primer sequences were listed in Table 1.

#### Inflammatory factors expression

It was well known that the levels of biomarkers IL-6 and MMP-13 reflect the degree of inflammation in chondrocytes and Col2a1 reflects the functional status of chondrocytes. To determine the protein expression levels, the supernatant of chondrocytes was analyzed by ELISA. Briefly, chondrocytes were seeded into 6-well plates with a density of 1×10^6^ per well. After 24 h of culture, the chondrocytes were attached and the medium was removed. Next, chondrocytes were incubated with PDA, PDA-Pd, and PDA-Pd plus NIR irradiation (0.8 W/cm^2^, 5 min) respectively at the same concentration of 50 μg/mL for 24 h, after IL-1β stimulation (10 ng/mL, 24 h), At last, the supernatant of all groups were collected and evaluated by IL-6, MMP-13, and Col2a1 ELISA kit (Solarbio, China) respectively according to manufacturer's instructions.

Finally, immunofluorescence staining was also executed to investigate the protein expression levels of chondrocytes. Details were: the treated chondrocytes were fixed with paraformaldehyde (Biosharp, China). After 15 min, the paraformaldehyde was removed and the culture wells were washed twice. After this, 100 μL 3% H_2_O_2_ solution was added to each culture well, and the H_2_O_2_ solution was removed after incubation at room temperature for 15 min, and the culture wells were washed twice. Then the treated chondrocytes were incubated with IL-6 polyclonal antibody (Proteintech, Wuhan, China), MMP-13 polyclonal antibody, and Collagen Type Ⅱ polyclonal antibody polyclonal antibody (Proteintech, Wuhan, China) respectively after blocking non-specific antibodies by goat serum (Beyotime, China). After incubating at 4°C for 12 h, chondrocytes were treated with secondary antibody FITC conjugated goat anti-rabbit IgG (H+L) (Boster, China) (1:100) for 1 h in the dark. At last, chondrocytes were incubated with a DAPI staining solution (Beyotime, China) for 5 min. Then the final images were collected.

#### Photothermal effects in vivo

For the *in vivo* photothermal effect detection of materials, four 8-week-old SD rats were bought from the Animal Research Committee of Guangxi Medical University. After the rats were anesthetized, 100 μL of PBS buffer, PDA solution (50 μg/mL), and PDA-Pd solution (50 μg/mL) were injected into the right knee joints of different rats. After 12 h, the rats were anesthetized again and then irradiated with NIR (808 nm, 0.8W/cm^2^) on the right knee joint. An FLIR NIR camera was used to capture the images and temperature values during NIR irradiation.

#### *In vivo* OA therapy

A total of 45 SD rats (male, 150∼180 g) were applied for *in vivo* experiments. The OA models were established via anterior cruciate ligament transection (ACLT) 4 weeks before further treatment and all rats were divided into 5 groups randomly: 1) Sham group: SD rats with only incising the skin and capsule of the knee joint, without further treatment; 2) OA group: OA models with intra-articular injection of PBS (0.2 mL); 3) OA + PDA group: OA models with intra-articular injection of PDA solution (50 μg/ml, 0.2 mL); 4) OA+PDA-Pd group: OA models with intra-articular injection of PDA-Pd solution (50 ug/mL, 0.2 mL); 5) OA+PDA-Pd+NIR group: OA models with intra-articular injection of PDA-Pd (50 ug/mL, 0.2 ml) and NIR irradiation. The intra-articular injection was implemented twice a week for 4 or 8 weeks, and NIR irradiation was implemented once per day with an intensity of 0.8 W/cm^2^ for 5 min.

Finally, rats were sacrificed by intraperitoneal injection of an overdose of sodium pentobarbital at pre-designed time points (4 and 8 weeks post-treatment). Immediately after euthanasia, 100 μL of PBS buffer was injected into the knee joints of rats, then the syringe was quickly withdrawn after mobilizing the knee joint of the rat to collect the synovial fluid. Subsequently, the knee joints and major organs (liver, spleen, heart, lung, kidney) were harvested. And the macroscopic observation of knee joints was scored according to Pelletier's macroscopic scoring methods with some modifications.^39^ In detail, the depth of the erosion of the articular cartilage was graded on a scale of 0 to 4, where 0 represented the normal surface, 1 represented the slight fibrillation or yellowish discoloration of the surface, 2 represented the erosion extending to the surface or middle layer, 3 represented the erosion extending to the deep layer, and 4 represented the erosion extending to the subchondral bone. By adding the grades of the medial condyle, the lateral condyle, the medial plateau, and lateral plateau, the macroscopic score of the total joint was obtained.^39^ Besides, the inflammatory factors (IL-6 and MMP-13) and Col2a1 contents of synovial fluid were measured by the ELISA kit (Solarbio, China), and the absorbance was measured at 450 nm. Finally, the harvested knee joints were fixed by 4% PFA for 2 days and decalcified in 0.5 M EDTA (pH= 7.2) for 1 month. Then the joint samples were embedded in paraffin and sectioned (5 μm thickness) for hematoxylin and eosin (H&E) staining, and Safranine O-fast green (Solarbio, China) staining. And the OA situation was evaluated according to the OARSI scoring system as described previously by three blinded observers.

At last, the major organs (liver, spleen, heart, lung, and kidney) were immersed in 4% PFA for fixation, embedded, sectioned, and then stained with H&E for observation.

#### The study of the toxicity of NPs.

A total of 12 SD rats (male, 150∼180 g) were used to detect the toxicity of the nanoparticles. All rats were randomly divided into the following four groups: 1) Control group: Intra-articular injection of PBS (0.2 mL). 2) PDA group: Intra-articular injection of PDA solution (50 ug/mL, 0.2 mL). 3) PDA-Pd group: Intra-articular injection of PDA-Pd solution (50 ug/mL, 0.2 mL). 4) PDA-Pd + NIR group: Intra-articular injection of PDA-Pd (50 μg/mL, 0.2 mL) and NIR irradiation. The intra-articular injection was implemented twice a week for 4 weeks, and NIR irradiation was implemented once per day with an intensity of 0.8 W/cm^2^ for 5 min.

Finally, at the pre-designed time point (after 4 weeks of treatment), the rats were fasted overnight prior to sampling. Then the rats were anesthetized by intraperitoneal injection of pentobarbital sodium. The venous blood was collected from the rats in all groups prior to sacrifice. Approximately 1.5 mL blood sample from each rat was collected into the tube containing heparin as the anticoagulant for the blood routine test (RBC, WBC, HGB, HCT, and PLT). And another 3 ml of venous blood was coagulated in a clean dry tube at room temperature for 1 h. Then the blood samples were centrifuged at 3500 rpm for 10 min at 4 °C, then the supernatant serum was collected for the blood biochemistry tests (AST, ALT, CREA, and urea).

### Statistical analysis

All statistical comparisons of means were performed using GraphPad Prism 8 software (GraphPad, USA). Multiple comparison tests were analyzed by one-way analysis of variance (ANOVA). (^∗^, ^#^ indicate p < 0.05; ^∗∗^, ^##^ indicate p < 0.01; ^∗∗∗^, ^###^ indicate p < 0.001; ns indicate not significant).

## Data Availability

This study did not report original code. Any additional information required is available from the lead contact up request.
