# Supplementary material for: Polydopamine-Pd nanozymes as potent ROS scavengers in combination with near-infrared irradiation for osteoarthritis treatment
Source: iScience. 2023 Apr 18;26(5):106605. doi: 10.1016/j.isci.2023.106605 (PMC10172781; doi:10.1016/j.isci.2023.106605)
Supplement: Document S1. Figures S1–S11 [file mmc1.pdf]

**iScience, Volume *26***

## Supplemental information

# Polydopamine-Pd nanozymes as potent ROS scavengers in combination with near-infrared irradiation for osteoarthritis treatment

**Hao Hu, Junxu Yang, Yanping Zhong, Jiawei Wang, Jinhong Cai, Cuijuan Luo, Zhiqiang Jin, Ming Gao, Maolin He, and Li Zheng**

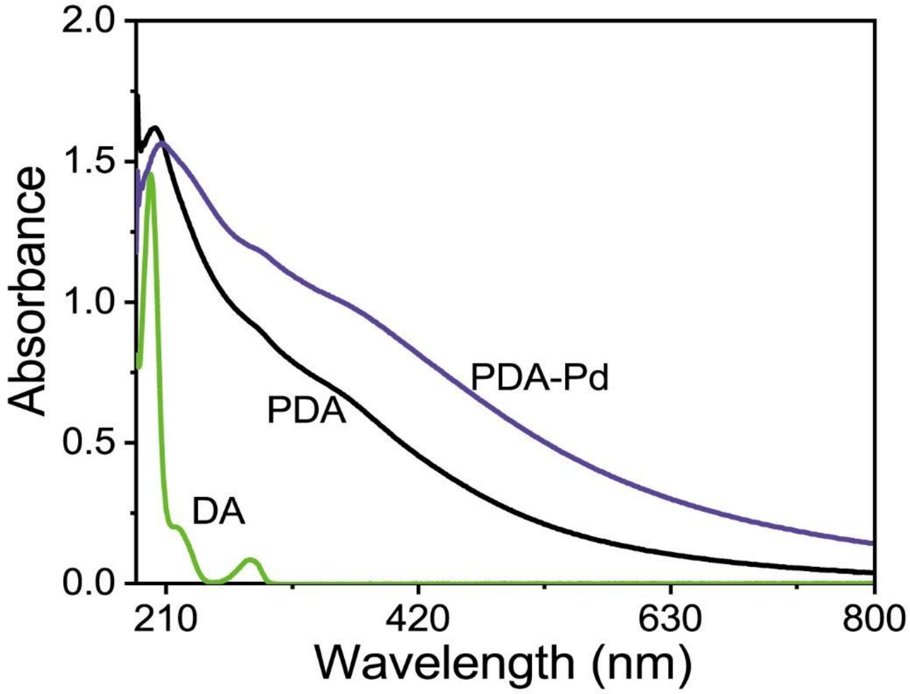


**Figure S1. UV-Vis spectra of DA, PDA, and PDA-Pd, Related to Figure 1**

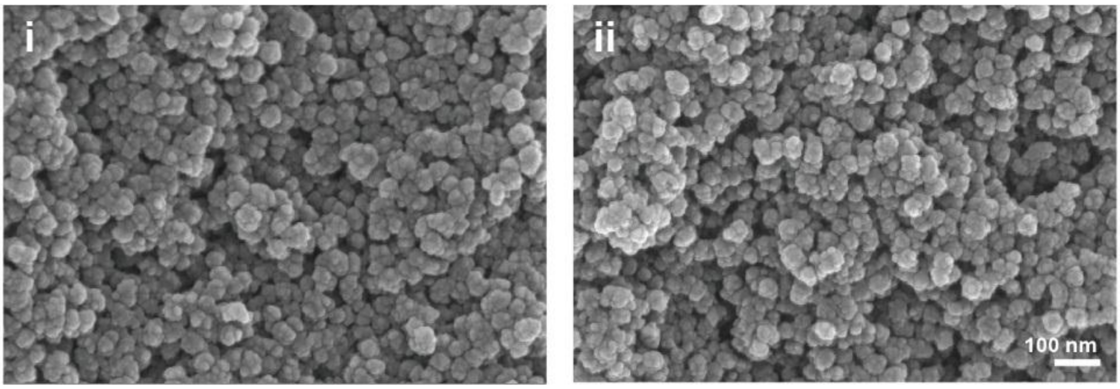


**Figure S2. SEM images of (i) PDA and (ii) PDA-Pd, Related to Figure 1**

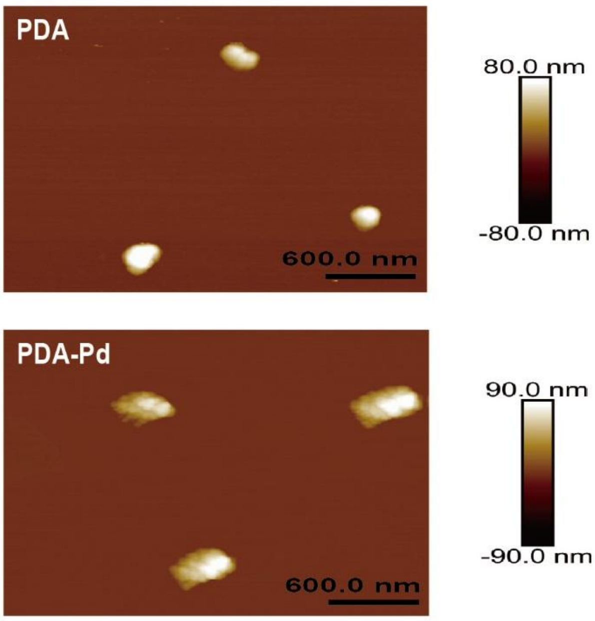


**Figure S3. AFM images of PDA and PDA-Pd, Related to Figure 1**

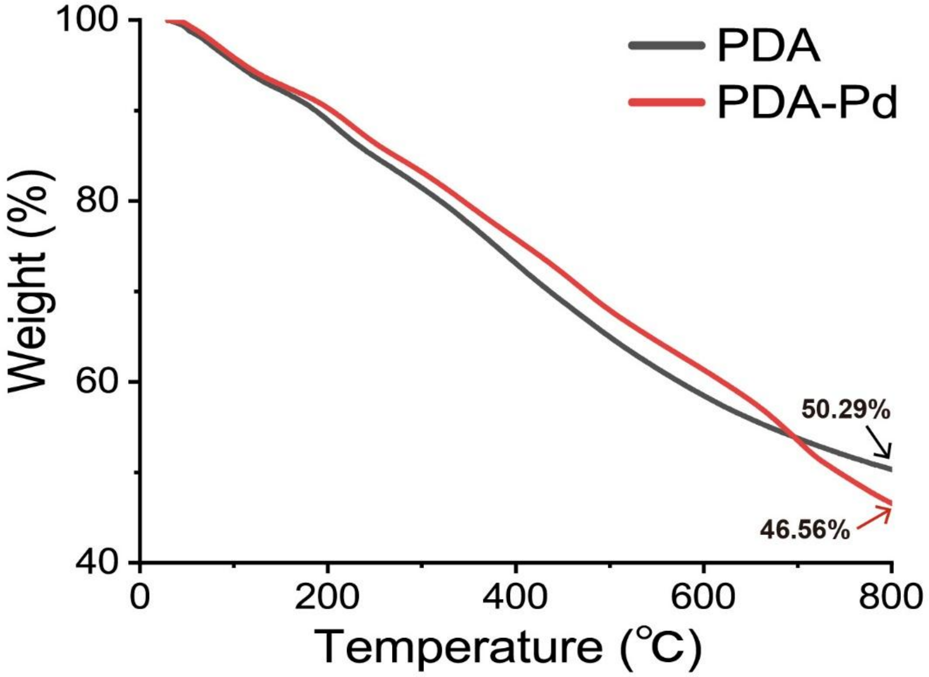


**Figure S4. TGA curves of PDA and PDA-Pd, Related to Figure 1**

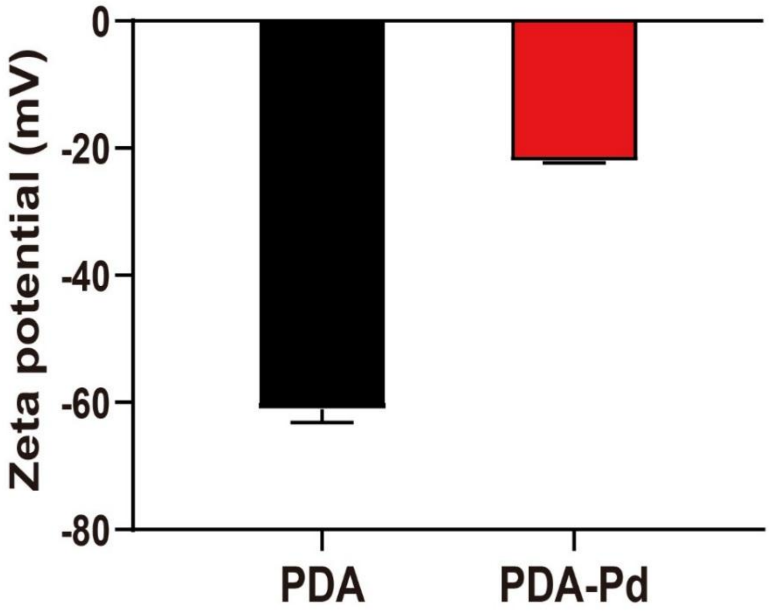


**Figure S5. Zeta potential of PDA and PDA-Pd in aqueous solution, Related to Figure 1**

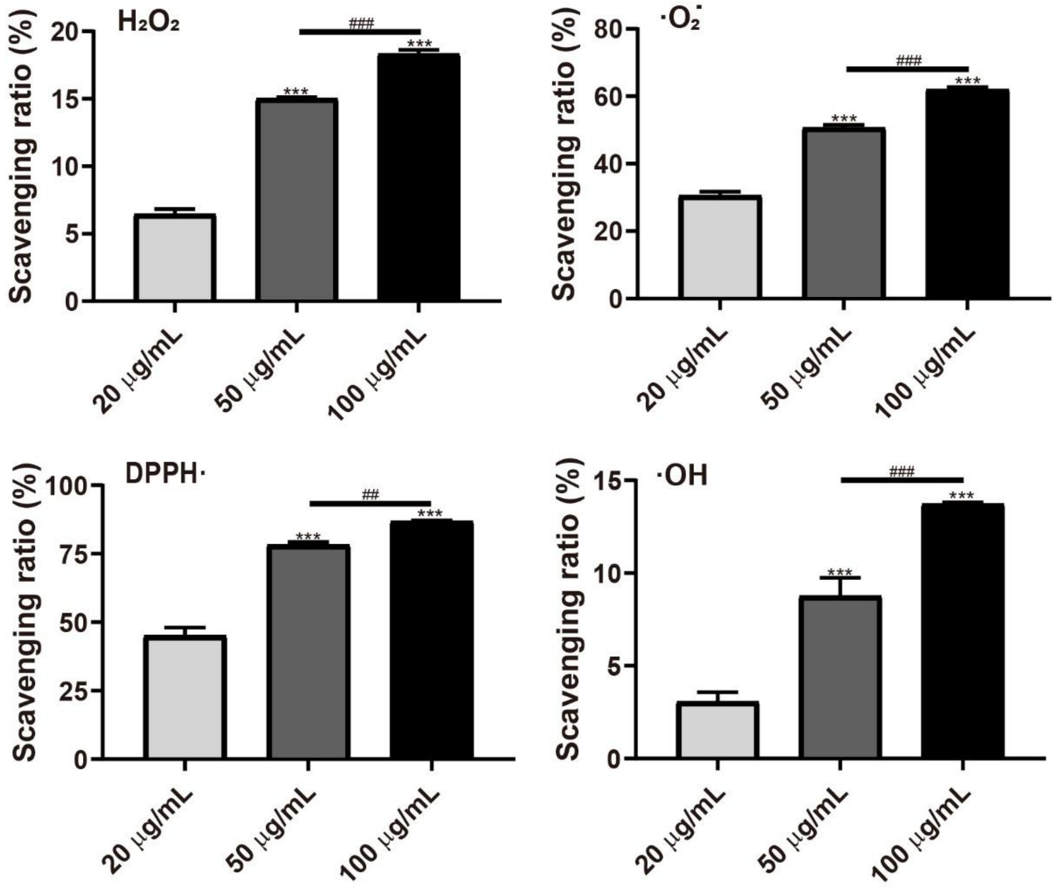


**Figure S6. $H_2O_2$, $\cdot O_2^-$, ·OH, and DPPH· scavenging ability of PDA-Pd at different concentrations, Related to Figure 3**

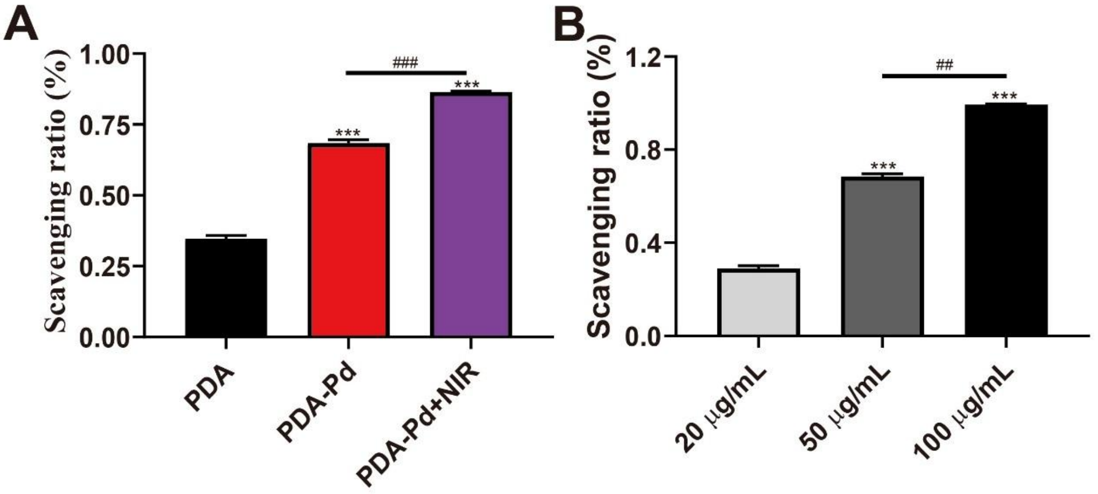


**Figure S7. The results of total ROS scavenging capacity, Related to Figure 3**

**(A)** Total ROS scavenging ability of PDA, PDA-Pd, and PDA-Pd plus NIR irradiation at the same concentration of 50 μg/mL. **(B)** Total ROS scavenging ability of PDA-Pd at different concentrations.

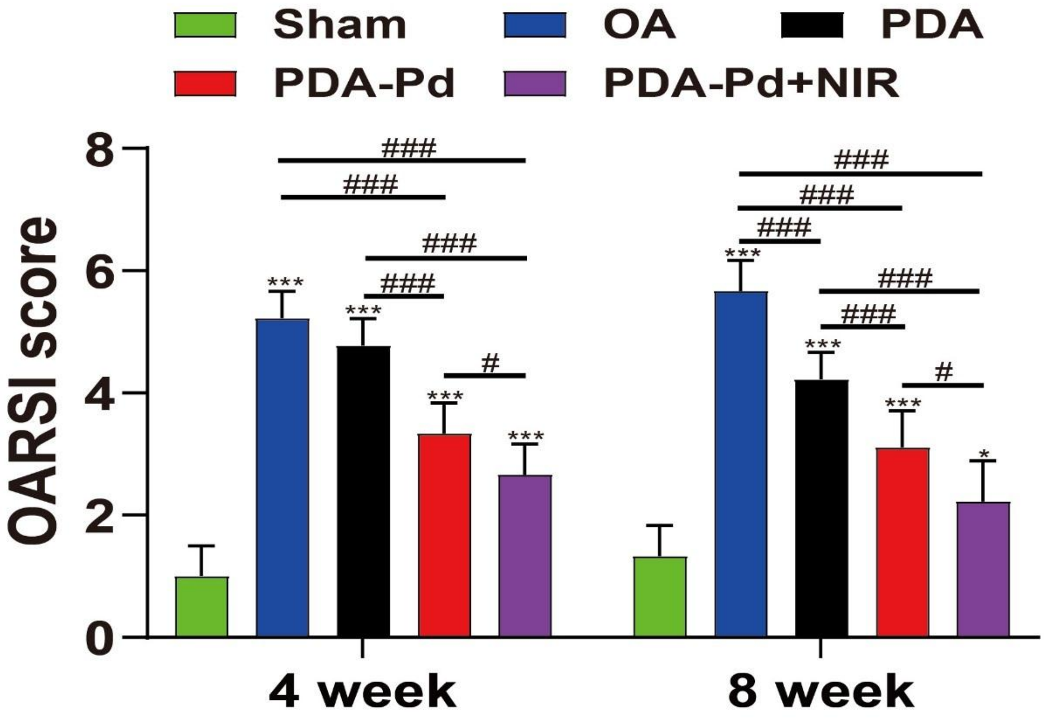


**Figure S8. OARSI scores of articular cartilage, Related to Figure 7** (n=3, values are the means ± SD. * symbol is for the comparison between the sham group and every other group, and # symbol is for the pairwise comparison. *, # indicates $P < 0.05$; **, ## indicates $P < 0.01$; ***, ### indicates $P < 0.001$).

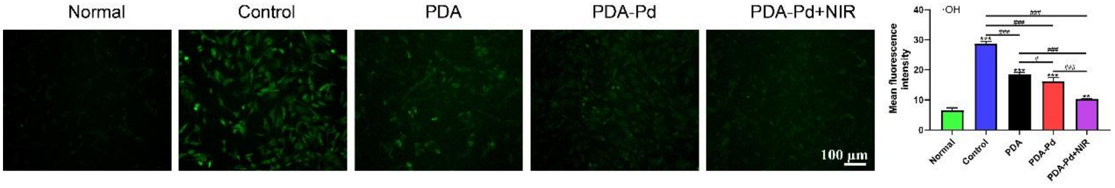


**Figure S9. The results of hydroxyl radical fluorescence staining, Related to Figure 5**
The hydroxy radical fluorescence staining (hydroxyphenyl fluorescein) of chondrocytes under different treatments. And quantification of mean fluorescence intensity in chondrocytes (Values are the means ± SD. * symbol is for the comparison between the normal group and every other group, and # symbol is for the pairwise comparison. * and # indicates P < 0.05; ** and ## indicates P < 0.01; *** and ### indicates P < 0.001).

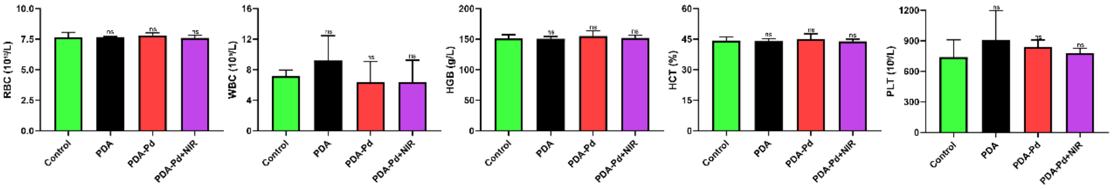


**Figure S10. The results of the blood routine test (RBC, WBC, HGB, HCT, and PLT), Related to Figure 7** (n=3, values are the means ± SD. the symbols above the error bars are for the comparison between the normal group and every other group. ns indicates no significant difference (P>0.05)).

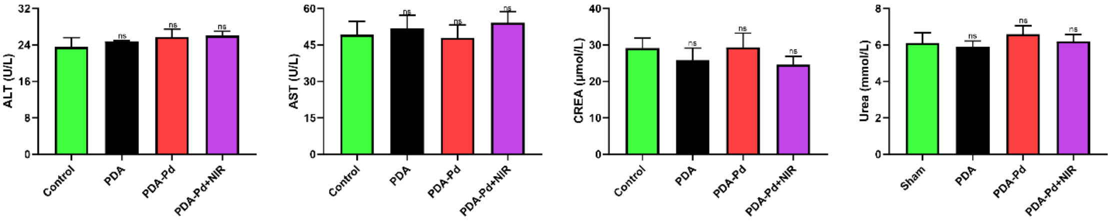


**Figure S11. The results of the blood biochemistry (ALT, AST, CREA, and urea), Related to Figure 7** (n=3, values are the means ± SD. the symbols above the error bars are for the comparison between the normal group and every other group. ns indicates no significant difference (P>0.05)).
